# Climate Change Drives Northwestward Migration of *Betula alnoides*: A Multi-Scenario MaxEnt Modeling Approach

**DOI:** 10.3390/plants14162539

**Published:** 2025-08-15

**Authors:** Yangzhou Xiang, Qiong Yang, Suhang Li, Ying Liu, Yuan Li, Jun Ren, Jiaxin Yao, Xuqiang Luo, Yang Luo, Bin Yao

**Affiliations:** 1 School of Geography and Resources, Guizhou Education University, Guiyang 550018, Chinaluoyang@gznc.edu.cn (Y.L.); 2 School of Biological Sciences, Guizhou Education University, Guiyang 550018, China; 3 Grasslands and Sustainable Farming, Production Systems Unit, Natural Resources Institute Finland, Halolantie 31A, Maaninka, FI-71750 Kuopio, Finland; 4 State Key Laboratory of Tree Genetics and Breeding, Institute of Ecology Conservation and Restoration, Chinese Academy of Forestry, Beijing 100091, China

**Keywords:** climate change, *Betula alnoides*, potential suitable habitat, MaxEnt model, centroid shift

## Abstract

Climate change poses unprecedented challenges to forest ecosystems. *Betula alnoides*, a tree species with significant ecological and economic value in southern China, has been the subject of studies on its distribution pattern and response to climate change. However, research on the distribution pattern of *B. alnoides* and its response to climate change remains relatively limited. In this study, we developed a MaxEnt model incorporating multiple environmental variables, including climate, topography, soil, vegetation, and human activities, to evaluate model performance, identify key factors influencing the distribution of *B. alnoides*, and project its potential distribution under various future climate scenarios. Species occurrence data and environmental layers were compiled for China, and model parameters were optimized using the ENMeval package. The results showed that the optimized model achieved an AUC value of 0.956, indicating extremely high predictive accuracy. The four key factors affecting the distribution of *B. alnoides* were standard deviation of temperature seasonality (Bio4), normalized difference vegetation index (NDVI), mean temperature of driest quarter (Bio9), and annual precipitation (Bio12). Among them, the cumulative contribution rate of climatic factors reached 68.9%, but the influence of NDVI was significantly higher than that of precipitation factors. The current suitable habitat of *B. alnoides* is mainly concentrated in the southwestern region, covering an area of 179.32 × 10^4^ km^2^, which accounts for 18.68% of China’s land area. Under the SSP126 scenario, the suitable habitat area first decreases and then increases in the future, while under the SSP370 and SSP585 scenarios, the suitable habitat area continues to shrink, with significant losses in high-suitability areas. In addition, the centroid of the suitable habitat of *B. alnoides* shows an overall trend of shifting northwestward. This indicates that *B. alnoides* is highly sensitive to climate change and its distribution pattern will undergo significant changes in the future. In conclusion, the distribution pattern of *B. alnoides* shows a significant response to climate change, with particularly prominent losses in high-suitability areas in the future. Therefore, it is recommended to strengthen the protection of high-suitability areas in the southwestern region and consider *B. alnoides* as an alternative tree species for regions facing warming and drying trends to enhance its climate adaptability.

## 1. Introduction

Climate change represents one of the most pressing challenges confronting global biodiversity, with species distribution shifts emerging as a critical ecological response requiring urgent investigation [1,2]. *Betula alnoides* Buch.-Ham. ex D. Don, a fast-growing deciduous broadleaf species endemic to southern China [3], exemplifies the vulnerability of regional flora to rapidly changing environmental conditions. Consequently, research on the climate change response of its distribution pattern is considered critically important. This species is widely distributed in tropical and subtropical montane forests across Yunnan, Guangxi, and Guizhou Provinces [4]. Its wood, characterized by a fine texture and moderate hardness, is highly valued in furniture manufacturing and construction industries. Furthermore, due to its strong ecological adaptability, *B. alnoides* is utilized for critical ecological restoration in erosion-prone areas, making it an indispensable component of local ecosystems. However, significant temperature increases, uneven precipitation distribution, and frequent extreme climatic events recently observed in southern China [5,6] are now posing severe challenges to its habitat. Climate change is known to directly affect plant distribution ranges, growth cycles, and ecological adaptability by altering temperature, precipitation patterns, and the frequency of extreme weather events [7,8]. Therefore, it is highly probable that the distribution pattern of *B. alnoides* will be profoundly altered. This vulnerability is exacerbated by the protracted life cycles of forest trees: *Betula alnoides*, with a life span exceeding 50 years, exhibits limited capacity for natural migration to track rapid climate shifts. Consequently, predicting its future distribution transcends theoretical inquiry, becoming an urgent conservation imperative to preempt irreversible population collapse. Our study directly addresses this challenge through integrated multi-driver modeling, providing actionable projections for climate-adaptive forest management.

Species distribution models (SDMs) have been extensively employed to investigate the relationships between species distributions and environmental factors [9,10]. Among these, the MaxEnt algorithm is frequently adopted owing to its robust predictive performance, particularly when data availability is limited [11]. Through the integration of climate data and species occurrence records, the MaxEnt model enables effective prediction of potential distribution ranges under current and future climate conditions, providing crucial references for conservation strategy development [12]. Although *B. alnoides* is acknowledged as ecologically significant in southern China, research on its distribution patterns and responses to climate change remains limited. Existing studies are primarily focused on areas such as breeding of superior varieties, forest cultivation, and pest and disease control [13,14,15,16]. However, the distribution dynamics of *B. alnoides* across China and its response mechanisms to climate change have not been comprehensively characterized. Furthermore, while SDMs have been extensively applied in ecological research, predictive studies specifically targeting the distribution of *B. alnoides* have not been reported. This gap is particularly notable concerning projected distribution shifts under future climate change scenarios. Therefore, to achieve a more comprehensive and accurate assessment of climate change impacts on the distribution pattern of *B. alnoides* in China, multiple factors were incorporated into the MaxEnt model construction for this study. In addition to climatic variables, the influences of topography, soil, vegetation, and human activities on its potential distribution were included. This integrated approach was adopted with the aim of more realistically reflecting the distribution dynamics of *B. alnoides* and its response mechanisms to environmental change.

The hypothesis of this study is that the distribution of *Betula alnoides* in China is highly sensitive to climate change, and its suitable habitat will significantly shift northwestward under future climate scenarios. The main objectives of this study are (1) to construct a MaxEnt model based on multiple environmental variables (including climate, topography, soil, vegetation, human activities, etc.) and the distribution data of *B. alnoides*, and to assess the predictive accuracy of the model, (2) to identify the dominant environmental factors influencing the suitable distribution of *B. alnoides* under current environmental conditions, (3) to predict the changes in the distribution pattern of *B. alnoides* under different future climate scenarios, and (4) to explore the potential impacts of climate change on the expansion or contraction of the distribution range of *B. alnoides*. Based on these research objectives, the following three research questions are proposed, and by addressing these questions, the mechanisms by which climate change affects the distribution pattern of *B. alnoides* will be revealed: (1) Is the distribution pattern of *B. alnoides* primarily influenced by climatic factors? (2) Are there significant differences in the impacts of topography, soil, vegetation, human activities, and other factors on its distribution? (3) Will the suitable distribution areas of *B. alnoides* shift toward higher latitudes or higher altitudes under future climate change scenarios? By answering these questions, this study will enhance our understanding of this key species’ response to climate change. It will also support decision-making in biodiversity conservation, ecosystem restoration, and adaptive forest management at the regional scale.

This study innovatively integrates multiple environmental variables, including climate, topography, soil, vegetation, and human activities, into the MaxEnt model to comprehensively assess the distribution dynamics of *B. alnoides* under climate change. It fills the gap in understanding how this species responds to future climate scenarios, providing a robust framework for predicting shifts in suitable habitats. The findings offer valuable insights for conservation strategies and adaptive forest management, enhancing the scientific basis for ecological restoration efforts.

## 2. Materials and Methods

### 2.1. Species Occurrence Data Collection and Preprocessing

**Figure 1:**
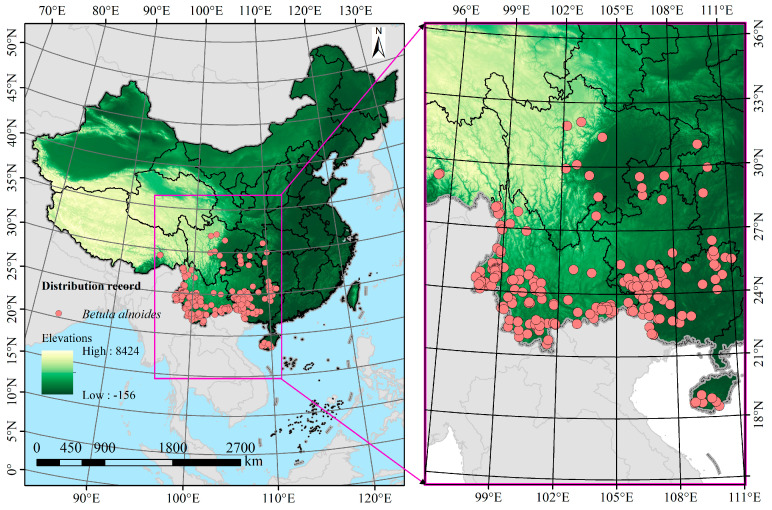
Geographical distribution of *B. alnoides* in China. The red dots represent individual occurrence records of *B. alnoides*.

The geographical distribution records of *B. alnoides* in China were mainly derived from the Chinese Virtual Herbarium (http://www.cvh.ac.cn/, accessed on 21 January 2025), the Global Biodiversity Information Facility (http://www.gbif.org/, accessed on 22 January 2025), and published academic literature retrieved from databases, including Web of Science (https://webofscience.clarivate.cn/, accessed on 25 January 2025), China National Knowledge Infrastructure (https://www.cnki.net/, accessed on 25 January 2025), and Google Scholar (https://scholar.google.com, accessed on 25 January 2025). Literature searches were performed on 16 March 2025 using the query string ‘(“*Betula alnoides*” OR “西南桦”) AND (distribution OR occurrence OR “potential habitat”)’ in Web of Science (Core Collection 1990–2025), CNKI (Chinese journals 1990–2025), and Google Scholar (first 200 hits). Only peer-reviewed articles or doctoral theses providing georeferenced coordinates were retained, yielding 41 papers after duplicate removal. During data collection, distribution records lacking precise latitude and longitude coordinates, where only town or village names were provided, were georeferenced using the “Jingweidu Query Positioning” (http://jingweidu.757dy.com/, accessed on 26 January 2025). Subsequently, the data were processed using Excel software, and duplicate records with identical coordinates were removed using the “Remove Duplicates” tool, resulting in a total of 251 valid and non-redundant distribution point records of *B. alnoides* within China. To eliminate the impacts of sampling bias and data redundancy on the prediction results, ArcGIS 10.8 was employed to filter the coordinate points. Using a spatial filtering method, only the distribution point closest to the center of each grid cell (with a spatial resolution of 2.5′ × 2.5′) was retained, yielding a final dataset of 206 distribution points for modeling (Figure 1). The data were then saved in .csv file as required by MaxEnt for subsequent analysis.

### 2.2. Environmental Variable Selection and Spatial Data Preparation

In this study, the influences of climate, topography, soil, vegetation, and human activities on the distribution of *B. alnoides* were comprehensively considered, and a total of 38 environmental variables were collected (Table S1). The 19 bioclimatic variables and elevation data were sourced from the global climate database WorldClim 2.1 (https://www.worldclim.org/, accessed on 16 June 2023). The topographic variables of slope and aspect were derived from the elevation data using ArcGIS 10.8 software. Soil data were obtained from the Harmonized World Soil Database v2.0 (https://www.fao.org/soils-portal/soil-survey/soil-maps, accessed on 28 January 2025). Human activities were represented by the human footprint index [17]. The normalized difference vegetation index (NDVI) data were derived from the MOD13A3 MODIS/Terra Vegetation Indices Monthly L3 Global 1 km SIN Grid V006 [18]. To accurately investigate the potential impacts of climate change on species distribution, future bioclimatic data for three time periods were selected: the 2050s (2041–2060), the 2070s (2061–2070), and the 2090s (2081–2100). Regarding the choice of climate models, we selected BCC-CSM2-MR after comparative benchmarking: it ranked top-three in CMIP6 for East Asia, showing the lowest temperature bias (−0.3 °C) and precipitation RMSE (0.9 mm d^−1^) versus ERA-Interim, outperforming CNRM-ESM2-1 and MIROC6 [19]. This study relied on this model and selected three Shared Socioeconomic Pathway (SSP) scenarios under it: SSP126, SSP370, and SSP585. Among them, SSP126 corresponds to a low-emission scenario, with an estimated global temperature increase of approximately 1.8 °C by 2100, SSP370 corresponds to a medium-emission scenario, with an estimated global temperature increase of approximately 2.7 °C by 2100, and SSP585 corresponds to a high-emission scenario, with an estimated global temperature increase of approximately 4.4 °C by 2100 [20]. The spatial resolution of all environmental factor layers was converted to 4.63 km × 4.63 km (2.5′ × 2.5′) using ArcGIS 10.8 software.

To mitigate the potential interference of high collinearity among environmental variables on the modeling results [21], the data for the 38 environmental variables (Table S1) and the geographic coordinates of the 206 occurrence points for *B. alnoides* were imported into Maxent 3.4.4 software [22] for preliminary modeling. During the modeling process, the random test percentage was set to 25%, the regularization multiplier to 1, and the number of replicates to 10, while other parameters were retained at their default settings. Concurrently, Pearson correlation analysis was performed on the environmental variables using SPSS 26.0 software to obtain the correlation coefficients among them (Table S2). We removed variables whose Pearson correlation coefficient |r| > 0.70 and lower contribution rates, retaining those with higher ecological relevance (e.g., Bio18 was retained despite |r| = 0.72 with Bio12 because it represents precipitation during the warmest quarter, a critical moisture window for *B. alnoides* seedling survival and growth that Bio12 alone cannot capture). Ultimately, 17 variables (Table S3) were retained for the final modeling analysis after this dual threshold screening.

### 2.3. MaxEnt Model Construction and Optimization

#### 2.3.1. Parameter Tuning Using ENMeval

Uncalibrated species distribution models may significantly deviate from the true distribution patterns due to probabilistic prediction biases [23], leading to inaccurate assessments of suitable habitat ranges for *B. alnoides* (either overestimation or underestimation) if directly applied to its potential distribution prediction. To address this issue, the MaxEnt model parameters were optimized using the R package ENMeval in this study [24,25], with a focus on two key parameters: regularization multiplier (RM: ranging from 0.5 to 4.0 with a step size of 0.5) and feature class (FC). Based on the five feature classes of linear (L), quadratic (Q), product (P), threshold (T), and hinge (H) functions [26], nine combinations of feature classes (FC) were constructed, including L, H, LQ, HPT, LQH, QHPP, QHPT, and LQHPT [27]. The model fit and complexity were evaluated using the Delta.AICc and OR10 metrics [28], and the optimal parameter combination was selected to drive the predictions, thereby ensuring the reliability of assessing the potential distribution of *B. alnoides* under climate change.

#### 2.3.2. Model Configuration and Cross-Validation

The optimized MaxEnt model was employed in this study to predict the potential suitable habitat distribution of *B. alnoides*. To ensure the precision and reliability of the model, a rigorous systematic modeling procedure was strictly followed: based on 206 valid occurrence points, the dataset was partitioned via stratified random sampling into a training set (155 points, 75%) and an independent testing set (51 points, 25%), which were used for model construction and validation, respectively. Key parameters were set in accordance with niche modeling standards: the cross-validation (Crossvalidate) repeated run mode was adopted, the maximum number of background points was set to 10,000 to adequately characterize environmental spatial heterogeneity, the regularization multiplier (RM) was fixed at 0.5 to balance model complexity and generalization ability, and the feature class (FC) was selected as the LQ combination to fit the ecological response curve of *B. alnoides*. Ten independent replicate runs were conducted to minimize stochasticity and ensure the stability of model output. The final prediction results were output as logistic probability values and saved in ASC format to meet the requirements for subsequent geospatial analysis.

#### 2.3.3. Model Performance Evaluation via AUC

The accuracy of the MaxEnt model was evaluated using the area under the curve (AUC) of the receiver operating characteristic (ROC) curve, true skill statistics (TSS), and Cohen’s kappa coefficient (Kappa) in this study. Generally, the higher the AUC, TSS, and Kappa value, the greater the predictive accuracy of the MaxEnt model [29]. The AUC value ranges from 0 to 1 [30,31], and its ecological significance is categorized as follows: an AUC value of 0.5–0.6 indicates model prediction failure, an AUC value of 0.6–0.7 suggests poor predictive accuracy, an AUC value of 0.7–0.8 indicates moderate predictive accuracy, an AUC value of 0.8–0.9 represents high predictive accuracy, and an AUC value of 0.9–1.0 signifies extremely high predictive accuracy [32,33]. Model performance based on TSS is categorized as poor (0.2–0.5), useful (0.6–0.8), or excellent (>0.8) [34]. Kappa thresholds are poor < 0.4, good 0.4–0.75, and excellent > 0.75 [35].

### 2.4. Habitat Suitability Classification and Mapping

To enhance the precision of the analysis, the MaxEnt model was run 10 times in this study, and the average value of the “maximum training sensitivity plus specificity logistic threshold (MTSPS)” was calculated and used as the threshold for delineating suitable and unsuitable areas [36], thereby reclassifying and visually presenting the suitable habitat map of *B. alnoides*. The MTSPS method is favored for its low sensitivity to species prevalence and background point ratios, and its robustness effectively reduces the risk of omission for low-prevalence species and the probability of misclassification for high-prevalence species, an advantage confirmed by Liu et al. [37] In the subsequent analysis, the output of the MaxEnt model was imported into ArcGIS 10.8 software, where the “Reclassify” tool was used in conjunction with the natural breaks (Jenks) method to classify the suitable habitat into four categories: unsuitable (0–0.1022), marginally suitable (0.1022–0.25), moderately suitable (0.25–0.45), and highly suitable (0.45–1.0).

### 2.5. Spatiotemporal Dynamics of Suitable Habitats Under Climate Scenarios

Under the influence of climate change, the spatial patterns of species’ suitable habitats are characterized by three types of changes: expansion, loss, and stability [30,38]. To intuitively compare the trends in the suitable habitat of *B. alnoides* under different climatic conditions across various future periods, the distribution results of the suitable habitats obtained from the MaxEnt model were first imported into ArcGIS 10.8 software. This step is essential because ArcGIS enables seamless integration and analysis of spatial data, crucial for visualizing and interpreting model outputs. It also offers advanced spatial analysis techniques like overlay analysis and thresholding, which are vital for delineating suitable and unsuitable habitats. Additionally, ArcGIS provides a robust platform for habitat suitability classification and mapping, enhancing our understanding of species distribution dynamics under different climate scenarios [39]. Subsequently, the ASC files of the predicted maps for different periods were converted into vector files, and a threshold value of 0.1022 was applied to transform the presence probability of *B. alnoides* into a binary format, thereby clearly delineating the distribution of suitable and unsuitable habitats in China. Thereafter, the overlay analysis tool in ArcGIS software was utilized to conduct an intersect analysis, generating a vector file of the overlap between the future predicted maps and the current predicted map. Finally, this vector file was converted back into a raster file to facilitate a clearer analysis of the trends in the retention, loss, and expansion of the potential suitable habitats of *B. alnoides*.

### 2.6. Centroid Migration Analysis of Suitable Habitat Range

The centroid locations of the potential suitable habitats of *B. alnoides* were calculated using SDMtoolbox v2.0 [40], and the migration distances under different climate change scenarios were quantified. Specifically, the study focused on the changes in the centroid locations of the suitable habitats of *B. alnoides* during the current period (1970–2020) and the 2050s, 2070s, and 2090s under three climate change scenarios: SSP126, SSP370, and SSP585. The procedure was as follows: first, the prediction results of the potential suitable habitats of *B. alnoides* obtained from the MaxEnt model were imported into ArcGIS 10.8 software and converted into binary vectors, with a threshold value of 0.1022 used to distinguish between suitable and unsuitable habitats; then, the geometric statistics function in the spatial analysis tools was employed, selecting the centroid geometric type to obtain the latitude and longitude coordinates of the centroid of the suitable habitats; finally, based on the coordinate data, the migration distances of the centroid of the suitable habitats between different scenarios were calculated.

## 3. Results

### 3.1. Model Optimization Outcomes and Predictive Accuracy

**Figure 2:**
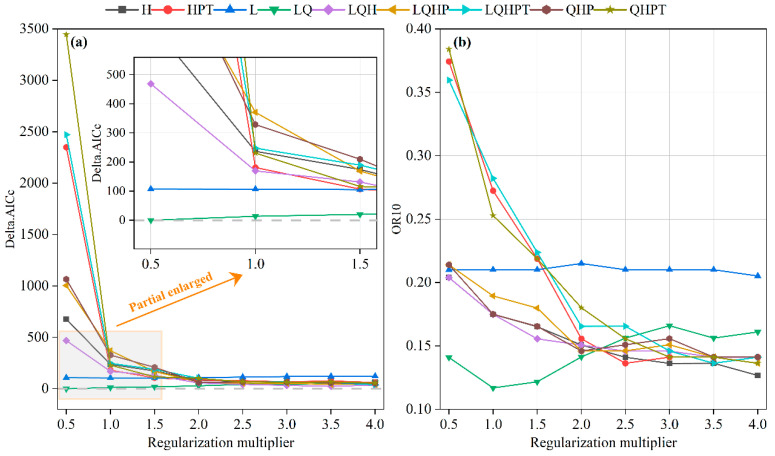
Evaluation metrics calculated by ENMeval for the species distribution model of *B. alnoides*. Feature complexity classes represented in the legend are L (linear), Q (quadratic), H (hinge), P (product), and T (threshold). (**a**) Delta.AICc and (**b**) OR10 derived from ENMeval.

The optimization effect of MaxEnt modeling parameters was evaluated using Delta.AICc and OR10 in this study [28]. When the default parameter configuration (RM = 1, FC = LQHP) was initially used, the Delta.AICc value of the model was as high as 370.8768 (Figure 2a). Anderson and Gonzalez [41] have pointed out that a Delta.AICc value exceeding 2 indicates inaccuracies in the MaxEnt model constructed with default settings, necessitating the selection of alternative parameters for modeling. Utilizing the ENMeval package, this study performed calculations on the geographic coordinates of 206 occurrence points of *B. alnoides* and the data of 17 important environmental variables, ultimately obtaining an effective parameter combination with Delta.AICc < 2: RM = 0.5/FC = LQ (Delta.AICc = 0), which satisfies the model selection criteria of the AICc information criterion. Further validation using the OR10 metric revealed that the OR10 value for the RM = 1/FC = LQHP combination was 0.1895, 34.45% higher than that for the RM = 0.5/FC = LQ combination (OR10 = 0.1409), indicating that the latter has superior predictive conservatism. Based on the principle of parsimony [42], the combination of RM = 0.5/FC = LQ was ultimately selected as the optimal parameter configuration in this study. This combination not only maintains the theoretically optimal value of Delta.AICc (Δ = 0) but also achieves the best balance between predictive accuracy and model complexity.

**Figure 3:**
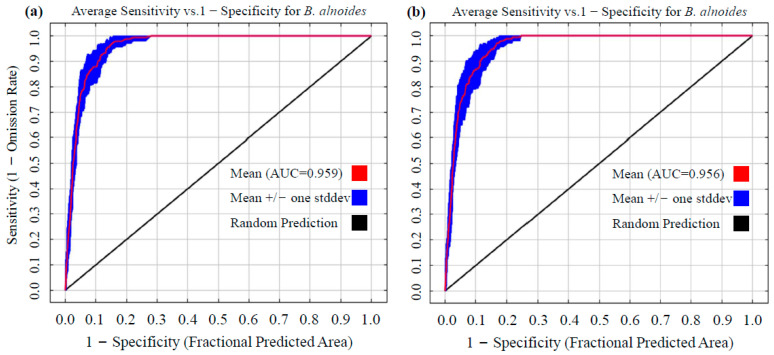
Validation of the MaxEnt model for *B. alnoides* using ROC analysis. (**a**) AUC with default settings and (**b**) AUC with refined parameters. (**a**) AUC from default parameters; (**b**) AUC from optimized parameters.

The modeling performance of the default parameter set (RM = 1, FC = LQHP) and the optimized parameter set (RM = 0.5, FC = LQ) was compared through systematic parameter validation in this study. The integrated analysis using 10-fold replicated cross-validation demonstrated that the AUC value for the default configuration was 0.959 (Figure 3a), while that for the optimized configuration was 0.956 (Figure 3b). Both values significantly exceeded the “excellent” discrimination threshold (AUC ≥ 0.9), confirming that the optimized model has a high explanatory power for species–environment interactions. It is worth noting that although the optimized model exhibited a marginal AUC decrease of 0.31% (ΔAUC = −0.003), the increase in regularization strength (RM = 0.5) and simplification of the feature class (FC = LQ) effectively mitigated the overfitting phenomenon observed under the default parameters. In addition, the optimized MaxEnt model achieved high predictive accuracy (TSS = 0.880 ± 0.006; Kappa = 0.638 ± 0.024), demonstrating exceptional ability to delineate *B. alnoides*’ current distribution in China.

### 3.2. Identification of Key Environmental Drivers of Species Distribution

**Figure 4:**
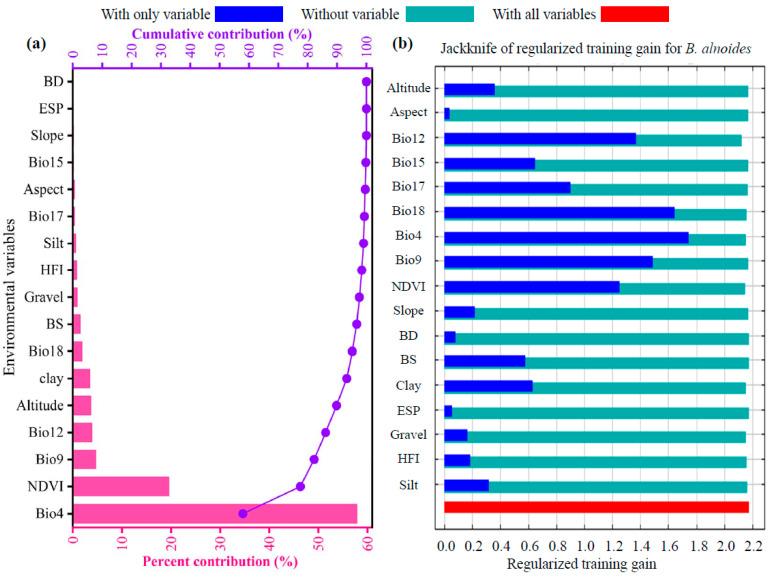
Contribution (**a**) and jackknife evaluation of regularized training gain (**b**) of environmental variables associated with *B. alnoides* via MaxEnt.

The optimized MaxEnt model was employed in this study to analyze the impact of 17 environmental variables on the potential geographical distribution of *B. alnoides*, using the contribution rate and jackknife test as evaluation criteria [43,44]. The top 10 environmental variables ranked by their contribution rates were as follows (Figure 4a): standard deviation of temperature seasonality (Bio4, 57.9%), normalized difference vegetation index (NDVI, 19.6%), mean temperature of driest quarter (Bio9, 4.7%), annual precipitation (Bio12, 3.9%), altitude (3.7%), clay fraction (clay, 3.5%), precipitation of warmest quarter (Bio18, 1.9%), base saturation (BS, 1.5%), gravel content (Gravel, 0.9%), and human footprint index (HFI, 0.8%). The 17 environmental variables can be categorized into five groups: climate, vegetation, soil, topography, and human activity, with cumulative contributions of 68.9%, 19.6%, 6.5%, 4.2%, and 0.8%, respectively. The normalized training gain from the jackknife test (Figure 4b) indicated that when used individually, the top six variables with the highest gains were standard deviation of temperature seasonality (Bio4), precipitation of warmest quarter (Bio18), mean temperature of driest quarter (Bio9), annual precipitation (Bio12), normalized difference vegetation index (NDVI), and precipitation of driest quarter (Bio17). Based on the criterion of cumulative contribution rate exceeding 85% [45,46], standard deviation of temperature seasonality (Bio4), normalized difference vegetation index (NDVI), mean temperature of driest quarter (Bio9), and annual precipitation (Bio12) were identified as the four key environmental variables, with a cumulative contribution rate of 86.1%, playing a significant regulatory role in the geographical distribution pattern of *B. alnoides* in China.

**Figure 5:**
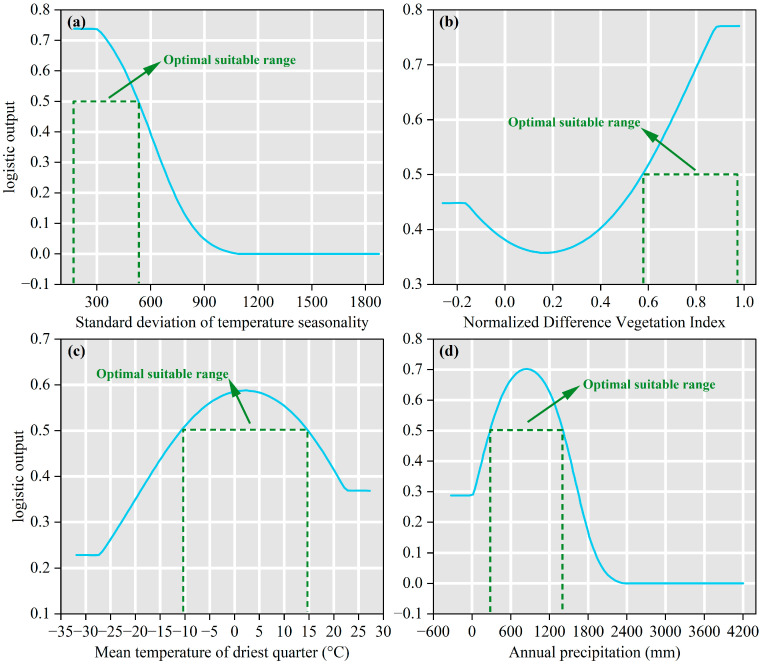
Curves for the presence probability of *B. alnoides* in response to variables. The sky-blue curves indicate the mean from 10 replicates, and horizontal forest-green dashed lines highlight the optimal suitable range. (**a**) Standard deviation of temperature seasonality; (**b**) Normalized difference vegetation index; (**c**) Mean temperature of driest quarter; (**d**) Annual precipitation.

To investigate the relationship between the presence probability of *B. alnoides* and the four key environmental variables, this study analyzed the single-factor response curves of the dominant variables (Figure 5). Specifically, the presence probability of *B. alnoides* remains stable at 0.74 when the standard deviation of temperature seasonality (Bio4) is between 172.25 and 300.48, and it drops sharply when Bio4 exceeds 300.48, reaching zero when it surpasses 1027.07 (Figure 5a). The normalized difference vegetation index (NDVI) shows constant presence probability when it is between −0.26 and −0.17, and between 0.89 and 0.98. When NDVI varies between −0.18 and 0.88, the presence probability first decreases, then increases, and decreases again, reaching a minimum at NDVI = 0.17 (Figure 5b). The mean temperature of driest quarter (Bio9) maintains a stable presence probability when it is between −31.88 °C and −27.36 °C, and between 22.12 and 27.27 °C. When Bio9 rises from −27.37 °C to 22.11 °C, the presence probability first increases and then decreases, with a maximum of 0.59 at 2.37 °C (Figure 5c). The presence probability of *B. alnoides* increases and then decreases as annual precipitation (Bio12) increases from 44.31 mm to 2215.20 mm, with a maximum of 0.70 at 910.76 mm, and it drops to zero when Bio12 exceeds 2215.20 mm (Figure 5d). The range of environmental variables corresponding to a presence probability of ≥0.5 in the response curves typically represents the optimal environmental conditions for species growth [47]. Therefore, the optimal suitable conditions for *B. alnoides* are Bio4 between 172.25 and 530.21, NDVI between 0.57 and 0.98, Bio9 between −10.43 °C and 14.41 °C, and Bio12 between 277.58 mm and 1415.40 mm (Figure 5).

In this study, the environmental variables used in the MaxEnt model, particularly the bioclimatic variables (BIOCLIM), are critical for understanding the ecological requirements of *B. alnoides*. These variables, such as the standard deviation of temperature seasonality (Bio4) and the normalized difference vegetation index (NDVI), are derived from climate data and represent key factors that directly influence the species’ survival, reproduction, and overall fitness. For instance, Bio4 reflects the variability in temperature throughout the year, which is crucial for the species’ phenological cycles and stress tolerance. NDVI, on the other hand, indicates the health and density of vegetation, which can affect competition and resource availability. By identifying these variables as significant predictors, our model provides insights into the specific climatic and ecological conditions that *B. alnoides* requires for its distribution. This biological interpretation enhances the ecological value of our species distribution model (SDM) results and supports targeted conservation and management strategies.

### 3.3. Current Distribution Pattern of Suitable Habitats for B. alnoides

**Figure 6:**
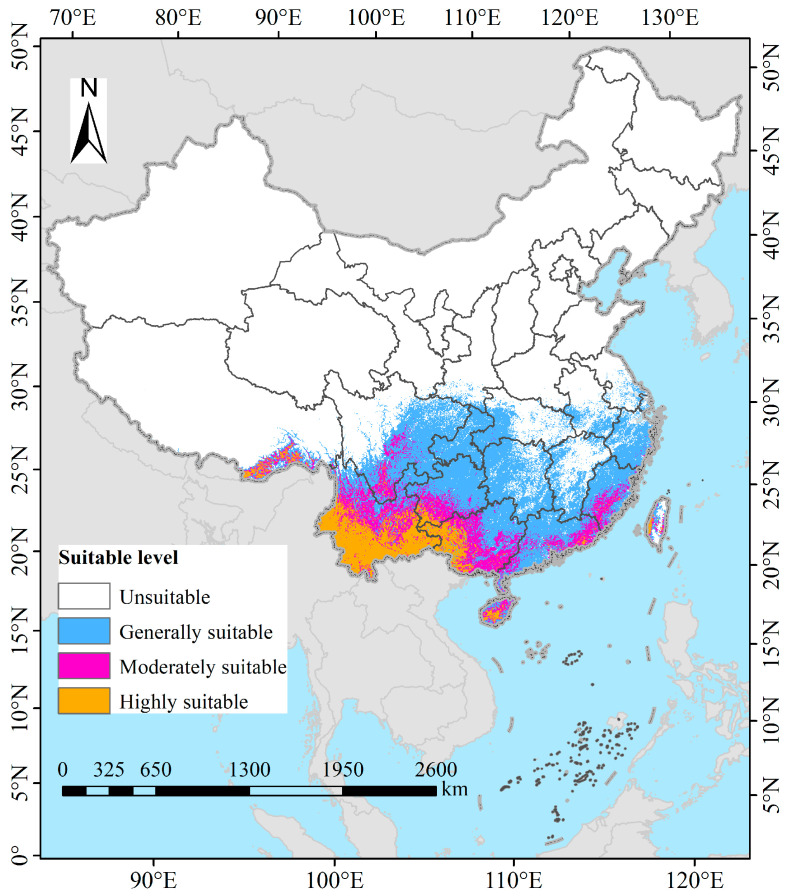
Potential distribution of *B. alnoides* in China under current climate scenarios.

Under current climatic conditions, the suitable habitats of *B. alnoides* in China are predominantly concentrated in the southern regions, with a total area reaching 1.7932 million km^2^ (equivalent to 179.32 × 10^4^ km^2^)—accounting for 18.68% of China’s territory (Figure 6). Specifically, high-suitability areas (26.10 × 10^4^ km^2^, 2.72%) are primarily distributed in Yunnan, Guangxi, Guizhou, and Hainan, while also occurring fragmentedly in Taiwan Province and Tibet Autonomous Region; concurrently, moderate-suitability areas (34.00 × 10^4^ km^2^, 3.54%) partially overlap these high-suitability areas and extend into Sichuan, Guangdong, and Fujian. Notably, low-suitability areas (119.21 × 10^4^ km^2^, 12.42%) are extensively distributed across southern China, Guangxi, Guangdong, Hunan, Jiangxi, Fujian, Zhejiang, and Shanghai, with scattered patches in Hainan, Taiwan, Hubei, Anhui, and Tibet, thereby demonstrating a tiered diffusion pattern from core southern habitats toward peripheral regions.

### 3.4. Projected Future Distribution Under Multiple Climate Scenarios

**Figure 7:**
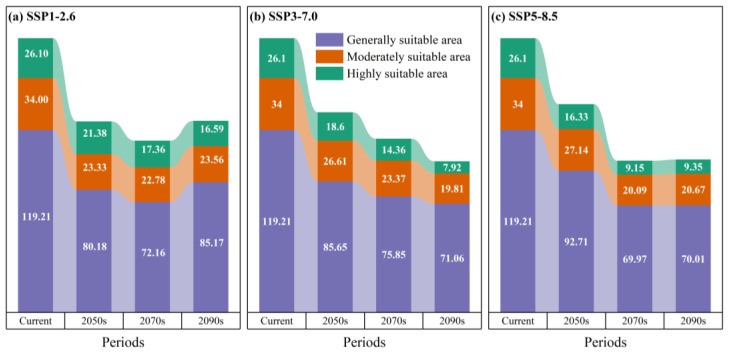
*B. alnoides* suitable habitat projections under climate scenarios (current to 2090s; unit: 10^4^ km^2^).

**Figure 8:**
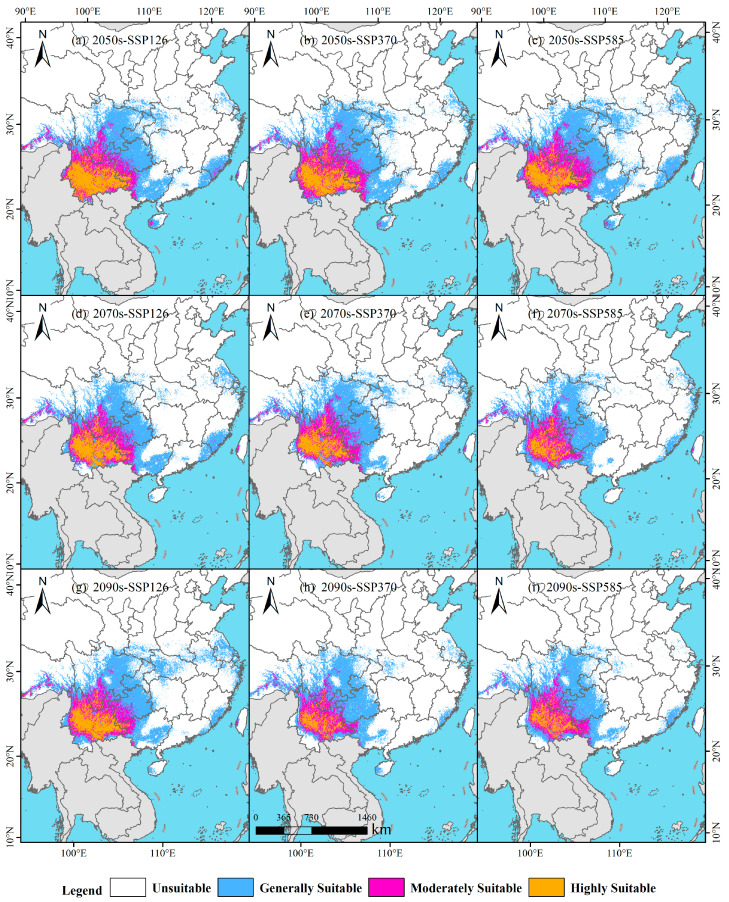
Potential distribution of *B. alnoides* in China under future climate scenarios.

Under the SSP126 scenario, *B. alnoides* suitable habitats exhibit phased changes (Figure 7 and Figure 8), where a total area of 124.89 × 10^4^ km^2^ is projected for the 2050s. High-suitability areas (21.38 × 10^4^ km^2^, 2.23% of China’s land area) are predominantly observed in Yunnan Province, while moderate-suitability areas (23.33 × 10^4^ km^2^, 2.43%) are distributed across Yunnan and Guizhou, with low-suitability areas (80.18 × 10^4^ km^2^, 8.35%) extensively covering Sichuan, Guizhou, Chongqing, Guangxi, and Hainan (Figure 7a and Figure 8a). By the 2070s, the total suitable area declines to 112.30 × 10^4^ km^2^, in which high-suitability areas (17.36 × 10^4^ km^2^, 1.81%) remain concentrated in Yunnan, moderate areas (22.78 × 10^4^ km^2^, 2.37%) encompass Yunnan, Guizhou, and Sichuan, and low-suitability areas (72.16 × 10^4^ km^2^, 7.52%) shift to Guangxi, Guizhou, Chongqing, Fujian, Jiangsu, Shanghai, and Sichuan (Figure 7a and Figure 8d). Entering the 2090s, a rebound to 125.32 × 10^4^ km^2^ is recorded, featuring persistently concentrated high-suitability areas in Yunnan (16.59 × 10^4^ km^2^, 1.73%), moderate areas (23.56 × 10^4^ km^2^, 2.45%) spanning Yunnan, Sichuan, and Guizhou, along with expanded low-suitability areas (85.17 × 10^4^ km^2^, 8.87%) across Hainan, Guangxi, Guizhou, Chongqing, Sichuan, Jiangsu, and Shanghai, plus fragmented occurrences in Yunnan, Fujian, Anhui, and Henan (Figure 7a and Figure 8g). Collectively, a dip–rebound trajectory is demonstrated for total suitable area under SSP126, accompanied by initial contraction then expansion in low/moderate areas, whereas high-suitability areas undergo progressive reduction.

Under the SSP370 scenario, *B. alnoides* suitable habitats demonstrate progressive contraction (Figure 8), where a total area of 130.86 × 10^4^ km^2^ is projected for the 2050s. High-suitability areas (18.60 × 10^4^ km^2^, 1.94%) are predominantly located in Yunnan, while moderate-suitability areas (26.61 × 10^4^ km^2^, 2.77%) maintain distribution patterns identical to SSP126 yet exhibit area expansion; concurrently, low-suitability areas (85.65 × 10^4^ km^2^, 8.92%) are concentrated in Guangxi, Guizhou, Sichuan, Chongqing, and Hainan (Figure 7b and Figure 8b). By the 2070s, total suitable area declines to 113.58 × 10^4^ km^2^, with high-suitability areas (14.36 × 10^4^ km^2^, 1.50%) remaining Yunnan-centric, moderate areas (23.37 × 10^4^ km^2^, 2.43%) distributed across Guizhou, Sichuan, Yunnan, and Guangxi (Figure 7b and Figure 8e), and low-suitability areas (75.85 × 10^4^ km^2^, 7.90%) primarily covering Guizhou, Yunnan, Sichuan, Guangxi, and Jiangsu. Entering the 2090s, a further reduction to 98.79 × 10^4^ km^2^ is observed, featuring substantially diminished high-suitability areas in Yunnan (7.92 × 10^4^ km^2^, 0.83%), moderate areas (19.81 × 10^4^ km^2^, 2.06%) confined to Yunnan and Sichuan, alongside low-suitability areas (71.06 × 10^4^ km^2^, 7.40%) predominantly aggregated in southwestern China (Figure 7b and Figure 8h). Collectively, a monotonic decline in total suitable area is recorded under SSP370, whereas all suitability classes exhibit progressive area reduction throughout the projection period.

Under the SSP585 scenario, *B. alnoides* suitable habitats exhibit phased shifts (Figure 8), where a total area of 136.19 × 10^4^ km^2^ is projected for the 2050s. High-suitability areas (16.33 × 10^4^ km^2^, 1.70%) are primarily concentrated in Yunnan Province, while moderate-suitability areas (27.14 × 10^4^ km^2^, 2.83%) span Guizhou, Yunnan, and Sichuan, with low-suitability areas (92.71 × 10^4^ km^2^, 9.66%) extensively distributed across Hainan, Guangxi, southwestern China, and southeastern coastal regions (Figure 7c and Figure 8c). By the 2070s, total suitable area contracts markedly to 99.22 × 10^4^ km^2^, characterized by high-suitability areas (9.15 × 10^4^ km^2^, 0.95%) now occurring in Yunnan and Sichuan, moderate areas (20.09 × 10^4^ km^2^, 2.09%) encompassing Yunnan, Sichuan, and Tibet, alongside low-suitability areas (69.97 × 10^4^ km^2^, 7.29%) predominantly occupying Yunnan, Sichuan, Guizhou, Chongqing, Guangxi, and southeastern coastal provinces (Figure 7c and Figure 8f). Entering the 2090s, a marginal recovery to 100.03 × 10^4^ km^2^ is observed, featuring persistent high-suitability concentration in Yunnan (9.35 × 10^4^ km^2^, 0.97%), moderate areas (20.67 × 10^4^ km^2^, 2.15%) confined to Yunnan and Sichuan, and low-suitability areas (70.01 × 10^4^ km^2^, 7.29%) distributed through Guizhou, Sichuan, Yunnan, Guangxi, and Fujian (Figure 7c and Figure 8i). Collectively, a contraction–recovery trajectory is demonstrated for total suitable area under SSP585, whereas all suitability classes display an initial reduction followed by subsequent expansion.

### 3.5. Temporal Trends in Habitat Stability, Loss, and Expansion

**Figure 9:**
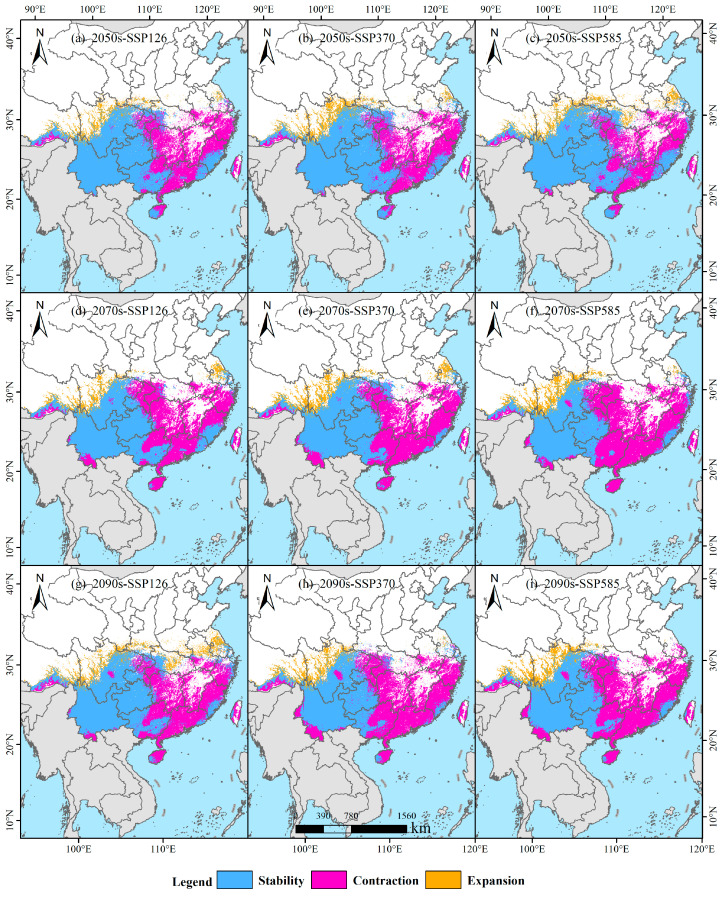
Future habitat stability, loss, and gain for *B. alnoides*.

Under the SSP126 scenario, *B. alnoides* habitat dynamics exhibit a phased pattern over time (Table 1; Figure 9). In the 2050s, retained habitats are projected to cover 136.22 × 10^4^ km^2^ (58.08%), with gained habitats at 15.71 × 10^4^ km^2^ (6.70%) and lost habitats at 82.60 × 10^4^ km^2^ (35.22%). By the 2070s, retained habitats decrease to 119.92 × 10^4^ km^2^ (51.21%), while gained habitats slightly decline to 15.26 × 10^4^ km^2^ (6.52%), and lost habitats increase to 98.98 × 10^4^ km^2^ (42.27%). In the 2090s, retained habitats recover to 128.04 × 10^4^ km^2^ (52.70%), accompanied by an increase in gained habitats to 24.21 × 10^4^ km^2^ (9.97%), while lost habitats decrease to 90.69 × 10^4^ km^2^ (37.33%). Overall, both retained and gained habitats show an initial decline followed by recovery under SSP126, whereas lost habitats follow an inverse trend—initial expansion followed by contraction.

Under the SSP370 scenario, progressive changes in *B. alnoides* habitat distribution are projected across future time periods (Table 1; Figure 9). In the 2050s, retained habitats are expected to cover 138.80 × 10^4^ km^2^ (58.00%), with gained habitats at 20.55 × 10^4^ km^2^ (8.59%) and lost habitats at 79.95 × 10^4^ km^2^ (33.41%). By the 2070s, retained habitats decline to 115.76 × 10^4^ km^2^ (48.26%), while gained habitats slightly increase to 20.86 × 10^4^ km^2^ (8.70%), and lost habitats expand to 103.25 × 10^4^ km^2^ (43.04%). In the 2090s, retained habitats further decrease to 102.59 × 10^4^ km^2^ (43.47%), accompanied by a reduction in gained habitats to 17.18 × 10^4^ km^2^ (7.28%) and an increase in lost habitats to 116.25 × 10^4^ km^2^ (49.25%). Overall, retained habitats show a continuous decline throughout the projection period, while gained habitats initially increase and then decrease. In contrast, lost habitats exhibit consistent expansion over time.

Under the SSP585 scenario, distinct patterns of *B. alnoides* habitat dynamics are projected for the 2050s (Table 1; Figure 9), with retained habitats accounting for 143.69 × 10^4^ km^2^ (59.72%), gained habitats for 12.85 × 10^4^ km^2^ (9.08%), and lost habitats for 75.09 × 10^4^ km^2^ (31.21%). By the 2070s, retained habitats decline sharply to 100.54 × 10^4^ km^2^ (42.16%), while gained habitats decrease slightly to 19.63 × 10^4^ km^2^ (8.23%) and lost habitats increase to 118.32 × 10^4^ km^2^ (49.61%). In the 2090s, retained habitats show a slight recovery to 102.02 × 10^4^ km^2^ (42.87%), whereas gained habitats continue to decline to 19.08 × 10^4^ km^2^ (8.02%). Meanwhile, lost habitats decrease marginally to 116.88 × 10^4^ km^2^ (49.11%). Overall, retained habitats follow a contraction–rebound trend, gained habitats show a consistent decline, and lost habitats exhibit an initial expansion followed by a slight contraction throughout the projection period.

### 3.6. Directional Shifts in the Geographic Centroid of Suitable Habitat

**Figure 10:**
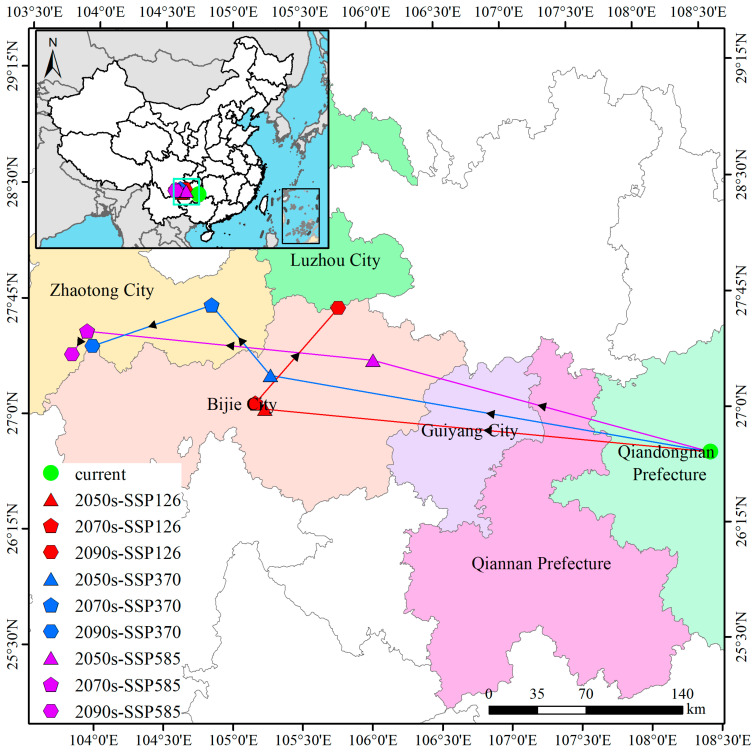
Shifts in the geographic centroid of suitable habitat for *B. alnoides* across climate scenarios.

Through range centroid analysis, habitat shifts of *B. alnoides* are quantified across future periods (2050s, 2070s, and 2090s) under various SSP scenarios (Figure 10). The current centroid (108.48° E, 26.70° N) is located in Gedong Town, Jianhe County, Guizhou. Under SSP126, the centroid is projected to migrate northwestward to Shendongguan Township, Nayong County (105.23° E, 27.03° N; migration distance ~324.56 km) by the 2050s, then to Weixin Town (105.17° E, 27.08° N; migration distance ~8.06 km) in the 2070s, before shifting northeast to Longchangying Town, Qixingguan District (105.78° E, 27.68° N; migration distance ~91.39 km) in the 2090s. Conversely, under SSP370, northwestward migration to Yachi Town, Qixingguan District (105.27° E, 27.25° N; migration distance ~323.87 km) is observed by the 2050s, followed by relocation to Muzhuo Town, Zhenxiong County, Yunnan (104.83° E, 27.72° N; migration distance ~66.40 km) in the 2070s, and a subsequent southwest shift to Zhongshui Town, Weining County (103.97° E, 27.45° N; migration distance ~91.43 km) in the 2090s. Under SSP585, the primary displacement is northwestward to Yuan Village, Jinsha County (106.03° E, 27.35° N; migration distance ~253.49 km) by the 2050s, with further migration to Luozehe Town, Yiliang County, Yunnan (103.92° E, 27.53° N; migration distance ~208.79 km) in the 2070s, culminating in a southwest shift to Taiping Subdistrict, Zhaoyang District (103.82° E, 27.38° N; migration distance ~19.93 km) by the 2090s. Under SSP126, the centroid initially migrates 324 km northwest (2050s) driven by rapid warming in Guangxi/Guizhou, then rebounds 91 km northeast (2090s) as high-elevation refugia in northern Yunnan become newly suitable. In contrast, SSP370/SSP585 show persistent westward drift (≈90 km) toward the cooler, wetter Yunnan-Guizhou Plateau edge, reflecting amplified temperature seasonality (Bio4) and declining precipitation in the eastern range that exceed the tolerance thresholds of *B. alnoides*.

## 4. Discussion

Unlike previous least-concern taxa, *B. alnoides* is a keystone subtropical pioneer whose climate sensitivity was undocumented. By integrating multi-factor parameter optimization with CMIP6 ensembles, we provide the first quantitative forecast of >480 km northwestward habitat shifts under warming, offering a transferable framework for climate-adaptive forestry of economically important but under-studied broadleaves.

### 4.1. Advances in MaxEnt Model Optimization and Its Ecological Implications

Model performance of MaxEnt was systematically optimized to enhance prediction reliability. Specifically, regularization multiplier (RM = 0.5) and feature classes (FC = LQ) were tuned via the ENMeval package, resulting in Delta.AICc being minimized to the theoretical optimum of 0—thereby satisfying the model selection criterion (ΔAICc < 2) proposed by Anderson and Gonzalez [41]. Concurrently, the OR_10_ statistic was reduced by 34.45% compared to default parameters, effectively mitigating overfitting risks [28]. For model validation, the approach by Kass et al. [24] was adopted, yielding an AUC value of 0.956. Although this represents a marginal 0.31% reduction relative to default parameters (AUC = 0.959), model generalizability was enhanced through simplified feature combinations (LQ) [21]. Furthermore, spatial filtering through 2.5′ × 2.5′ grids was implemented to reduce sampling bias, while the MTSPS threshold method [37] was applied to decrease omission rates for low-prevalence species, thus ensuring habitat classification accuracy. Despite 2.5′ filtering, uneven survey effort and road-accessible sampling may over-represent Yunnan/Guangxi, potentially inflating the modeled suitability there and underestimating northern limits. To mitigate this, we employed spatially biased background points weighted by human footprint and verified robustness with k-fold block CV (AUC 0.93–0.96), confirming that predictions remain reliable under residual bias. The final AUC exceeding 0.95 surpassed the “exceptional” benchmark established by Swets [33]. Collectively, these optimizations maintained high predictive accuracy while achieving optimal balance between model complexity and conservatism [25].

Compared with previous studies, our research has made significant progress in the depth of model parameter optimization. Specifically, it has broken through the limitations of traditional reliance on default parameters. For example, Shen et al. [48] directly used RM = 1 when modeling *Castanopsis hystrix* Miq., whereas this study, for the first time, achieved a zero-deviation parameter combination for *B. alnoides* (RM = 0.5/FC = LQ) [26]. In terms of overfitting control, this study quantified the trade-off between feature complexity and predictive conservatism (a ΔAUC = −0.003 in exchange for a 34.45% reduction in OR10), which is more operational than the qualitative suggestions of Radosavljevic and Anderson [21]. Regarding threshold selection, the maximum training sensitivity plus specificity (MTSPS) was used instead of the commonly used 10th percentile [49,50], effectively addressing the misjudgment of low-suitability areas [51,52]. Collectively, this methodology establishes a novel paradigm for modeling the potential distribution of deciduous broadleaf tree species in Southern China, particularly for those with sparse occurrence records.

### 4.2. Dominant Environmental Factors Shaping the Distribution of B. alnoides

The dominant regulatory mechanisms for *B. alnoides* habitat were identified through jackknife tests and percentage contributions of environmental variables [30,53]. Specifically, the curves reveal clear ecological optima: high probability occurs when Bio4 (temperature seasonality) is moderate (172–530), NDVI is ≥0.57, Bio9 (mean temperature of the driest quarter) is −10 to 14 °C, and Bio12 (annual precipitation) is 278–1415 mm. These thresholds align with the species documented preference for montane subtropical forests with moderate climate variability and abundant vegetation cover. The cumulative contributions of five environmental categories were ranked as climate (68.9%) > vegetation (19.6%) > soil (6.5%) > topography (4.2%) > human activities (0.8%). Notably, the low contribution of the human footprint index (HFI) suggested that current anthropogenic disturbance is not a limiting factor for the species’ potential distribution. This phenomenon was potentially attributable to (1) the difficulty of regional-scale models in precisely capturing localized disturbance signals (e.g., selective logging and road construction) affecting potential distribution [54,55], (2) the ecological adaptability of *B. alnoides*, as a pioneer species, to moderate disturbance levels [56,57], and (3) the lack of quantification standards for low-intensity agroforestry disturbances within the species’ range in generalized HFI datasets [58].

Compared to some ecologically similar tree species, *B. alnoides* was found to exhibit strong dependence on the standard deviation of temperature seasonality (Bio4), with a contribution rate as high as 57.9%. Convergence with *Podocarpus neriifolius* D. Don (Bio4 contribution: 66.3%) was observed [59], while a striking divergence from *C. hystrix* (Bio4 contribution: 0%) was highlighted [48]. This phenomenon reflects both convergent evolution and significant differentiation in the standard deviation of temperature seasonality adaptation among species occupying similar niches, potentially attributable to climatic differences in their native ranges leading to distinct evolutionary strategies. Regarding the NDVI influence on *B. alnoides* distribution, a contribution rate of 19.6% was recorded, significantly exceeding that observed in some widely distributed species; for instance, NDVI contributed merely 8.7% in *Pistacia chinensis* Bunge models [60]. This difference was potentially linked to *B. alnoides*’ pioneer species characteristics in degraded ecosystems, requiring higher vegetation cover for competitive maintenance [57]. In terms of precipitation dependence, the low contribution of Bio12 (3.9%) contrasted sharply with findings for *Sorbus alnifolia* (precipitation contribution > 37%) [61], further corroborating *B. alnoides*’ dry-season adaptability evidenced by its broad thermal tolerance. Furthermore, *B. alnoides*’ “strong temperature–weak precipitation” response pattern was noted to be similar to patterns reported for *Cinnamomum camphora* (Linn.), *Sapindus delavayi* (Franch.) Radlk., and *Jacaranda mimosifolia* D. Don. [62,63,64], yet distinct from the “strong precipitation–weak temperature” patterns characteristic of *Castanopsis carlesii*, *Camellia sinensis*, and *Betula luminifera* [65,66,67]. This divergence was potentially attributed to *B. alnoides*’ specific adaptation to dry, hot valley environments [68].

Based on the analysis of key environmental factors (cumulative contribution rate: 86.1%), three management implications were proposed: (1) Conservation priority planning for *B. alnoides* should be centered around the Bio4 threshold (below 530.21), with enhanced protection measures specifically targeted at high-suitability habitats where current Bio4 values fall between 280 and 450 (e.g., Yunnan province) [69]. (2) Ecological restoration efforts should be focused on areas with NDVI > 0.57 (e.g., southern Guizhou) [70], avoiding indiscriminate introduction in low vegetation cover areas (NDVI < 0.17). (3) For climate change adaptation, *B. alnoides*’ pioneer status and drought tolerance (Bio9 range: −31.88–27.27 °C) facilitate northwest migration, supporting its use as a climate-adaptive replacement species in warming–drying regions. However, its slow maturation (20–30 years) may lag behind rapid climate shifts, while local adaptations in dry, hot valley populations (e.g., deeper roots and waxy leaves) could enhance resilience in contracting habitats [57,71,72]. Collectively, key factors (Bio4, NDVI, and cumulative contribution: 86.1%) were successfully identified, and the dominance of climatic factors (68.9%) was effectively validated. However, the significant contribution of the vegetation factor (NDVI, 19.6%) was found to surpass that of precipitation factors, necessitating revision of the initial “purely climate-controlled” hypothesis. Quantitative results confirmed that among non-climatic factors, only NDVI exerted a significant influence (19.6%), while contributions from topography, soil, and human activities were minimal (0.8–6.5%).

### 4.3. Climate Change Impacts on Range Dynamics and Habitat Suitability

The response patterns of *B. alnoides* distribution to climate change were revealed through multi-scenario simulations. The current suitable area (179.32 ×10^4^ km^2^, 18.68% of China’s land area) was projected to decrease by 45–48% (98.79 × 10^4^ to 100.03 ×10^4^ km^2^) under SSP370 and SSP585 scenarios by the 2090s, with the highest-suitability habitat experiencing the most severe loss (69.7% reduction under SSP370). A persistent northwestward centroid shift was observed (from Jianhe County, Guizhou Province to Zhaoyang District, Yunnan Province), with a displacement distance of 482.21 km projected under SSP585 by the 2090s, supporting the “high-elevation refuge” hypothesis [73,74]. Scenario divergence was evident: under SSP126, a “V-shaped” fluctuation in suitable area was projected (increase of 130,000 km^2^ by the 2090s relative to the 2070s), while continuous contraction was projected under SSP370. This indicated that effective emission mitigation policies could significantly alleviate habitat loss [75,76]. During the shift, retention areas were concentrated on the Yunnan-Guizhou Plateau (e.g., >40% retention rate in Yunnan under SSP585), whereas expansion areas were primarily located along the northern edge of the Sichuan Basin (expansion rate ~1.8 × 10^4^ km^2^/decade). Collectively, these findings demonstrate that climate change—particularly intense warming under high-emission scenarios like SSP585—is profoundly reshaping the species’ suitable habitat range.

Compared with existing species distribution response studies, the methodological distinctiveness of this work is demonstrated through three key dimensions. Regarding migration velocity, the northwestward centroid shift rate of *B. alnoides* (2.5–5 km/year) was found to be slower than that of *Alnus cremastogyne* Burk. (3–10 km/year) [77], but faster than *Quercus baronii* Skan (1.1–1.3 km/year) [78]. The differences observed among the three studies may be attributed to variations in seed dispersal distances and interspecific competition pressure at migration fronts [79,80]. Regarding the vulnerability of high-suitability habitats, Yunnan Province, identified as the core suitable region for *B. alnoides*, is projected to experience a severe contraction of 70% in area under the moderate–high-emission scenario (SSP370). This reduction surpasses the habitat retention observed under SSP585, underscoring its heightened susceptibility to acute climatic stress. This outcome was likely driven by the intensified standard deviation of temperature seasonality under SSP370, which may adversely impact growth and reproduction: accelerated springtime warming could enhance soil moisture evaporation [81,82], rapid autumn cooling may compromise seed maturation and dispersal [83], and temperature fluctuations could disrupt physiological processes while reducing drought tolerance [84]. Expansion mechanisms were characterized by a 24 × 10^4^ km^2^ habitat gain by the 2090s under SSP126, though predominantly in low-suitability categories. Realizing the projected range shift depends critically on dispersal capacity. *B. alnoides* seeds are wind-dispersed and exhibit a median dispersal distance of 60–120 m under current wind regimes [79]. Centroid shifts of 2.5–5 km year^−1^ would, therefore, require 1200–2000 years by wind alone, far exceeding the 70-year projection window. Assisted migration via seed transfer and the creation of landscape corridors are, therefore, essential, and we recommend prioritizing planting along 50 km northwest transects to bridge the dispersal gap.

### 4.4. Quantifying Non-Climatic Drivers Through Model Integration

While field measurements of edaphic and topographic variables were beyond the scope of this modeling study, our MaxEnt outputs provide quantitative validation of their ecological roles. Topographic mediation through elevation (3.7% contribution) and slope/aspect (implicitly captured in DEM derivatives) primarily functioned as proxies for thermal moisture gradients, evidenced by the optimal elevation range (800–1500 m) in response curves aligning with observed distributions in Yunnan’s mountainous terrain, where cooler temperatures buffer climate extremes [85]. Soil constraints reflected *B. alnoides*’ preference for well-drained acidic soils (pH 4.5–6.0), with clay fraction (3.5%) and base saturation (1.5%) collectively explaining 5.0% of habitat suitability, as found in field studies [70], mechanistically consistent with the species’ sensitivity to waterlogging in Guangxi plantations [86]. Vegetation competition was empirically confirmed by NDVI’s high contribution (19.6%), where optimal values (0.57–0.98) correspond to closed-canopy forests, enabling *B. alnoides* to dominate the subcanopy layer [4,87]. Crucially, the minimal human footprint impact (0.8%) suggests that anthropogenic pressures within the species’ core range are currently subcritical. This could be attributed to the relative stability of human activities in southwest China [88]. These model-derived insights compensate for the absence of in situ measurements through integration of published ecological validations.

### 4.5. Study Limitations and Directions for Future Research

The limitations of this study are reflected in three aspects in terms of methodology and the interpretation of ecological mechanisms. Firstly, the spatial filtering employed to reduce sampling bias resulted in a final dataset of 206 occurrence points. While necessary to mitigate spatial autocorrelation and model overfitting, and despite the high model performance (AUC > 0.95), this sample size may limit the representation of fine-scale environmental heterogeneity across the species’ extensive range in China. Secondly, the simplification of interspecific interactions and biotic factors remained pronounced. Although the NDVI was incorporated to represent competition, dynamic influences of key competitors (e.g., *C. hystrix*, *Erythrophleum fordii* Oliv., and *Pinus kesiya* var. *langbianensis* (A. Chev.) Gaussen ex Bui) or symbiotic microorganisms were not quantified. Thirdly, inadequate representation of anthropogenic factors was observed: the human footprint index could only reflect macro-scale disturbance intensity, failing to capture microhabitat heterogeneity induced by regional agroforestry management or selective logging. This is particularly important in low-suitability areas where local interventions may alleviate climatic pressures; however, socioeconomic scenarios simulating such measures were not coupled with models. Fourthly, unmodeled biotic interactions may constrain shifts. The NDVI-driven suitability pattern (19.6% contribution) suggests competition suppression, evidenced by significantly lower presence probability at NDVI = 0.17 where shade-tolerant competitors dominate. This implies that predicted habitat gains in Sichuan Basin could be limited by extant vegetation. Fifthly, biological constraints on migration capacity were overlooked. Centroid shift projections assumed unrestricted colonization of newly suitable areas, but neither *B. alnoides*’ limited seed dispersal distance nor the absence of animal dispersal vectors was accounted for, and genetic diversity hotspots in Yunnan may enable adaptive evolution to seasonal instability (Bio4), though genomic validation is needed, potentially overestimating migration rates. Additionally, the MaxEnt model, despite its strengths, also has limitations, including sampling bias, assumption of variable independence, overfitting risk with high-dimensional data, and dependency on input data quality.

Future research should focus on the integration of multi-scale mechanisms and adaptive management. On the one hand, multi-model coupling and process-driven approaches should be conducted. Process-based models should be integrated with MaxEnt models to simulate the dynamic impacts of biological feedback, such as interspecific competition and disease spread, on distribution patterns. Human–nature coupled models should be developed, incorporating high-resolution land-use change scenarios to assess the potential of ecological restoration projects to enhance habitat connectivity. On the other hand, key biological mechanisms should be validated. Controlled experiments should be conducted to determine the climatic tolerance thresholds of *B. alnoides*, and functional traits should be analyzed to elucidate adaptive differences among populations. Physiological response parameters in the models should be calibrated accordingly. Landscape genetics should be integrated with species distribution models to identify migration corridors using genomic data and to assess the impacts of gene flow limitations on population persistence. In addition, based on the refuge areas identified in this study, refuge microhabitat maps should be drawn using microtopography and soil moisture retention data. Conservation prioritization algorithms should be employed to simulate the expansion of protected area networks under different emission reduction pathways, with a focus on optimizing the design of ecological corridors. Future research could also explore using the biomod2 framework to address some of the limitations of MaxEnt, such as sampling bias and variable interdependence. Biomod2 allows for ensemble modeling, which can improve prediction accuracy and robustness by combining multiple algorithms and accounting for data uncertainty.

## 5. Conclusions

The potential suitable habitat distribution of *B. alnoides* in China and its response to climate change were comprehensively analyzed in this study using the optimized MaxEnt model, which incorporated multiple environmental variables, including climate, topography, soil, vegetation, and human activities. The results indicated that the current suitable habitat of *B. alnoides* is primarily concentrated in the southern region, covering an area of 179.32 × 10^4^ km^2^, which accounts for 18.68% of the national land area. Under future climate scenarios, significant changes were observed in both the area and distribution pattern of the suitable habitat: under the SSP126 scenario, the suitable habitat area first decreased and then increased, whereas under the SSP370 and SSP585 scenarios, the suitable habitat area continuously shrank, with particularly substantial losses in the highly suitable areas. The centroid shift analysis revealed an overall northwestward migration of the suitable habitat, with migration distances varying significantly across different scenarios. By optimizing the MaxEnt model parameters, this study significantly improved the predictive accuracy and identified key environmental factors, such as standard deviation of temperature seasonality and normalized difference vegetation index, thereby revealing the sensitivity of *B. alnoides* to climate change. The findings not only provide a scientific basis for the conservation and ecological restoration of *B. alnoides* but also offer important decision-making support for adaptive forest management in response to climate change. In conclusion, we recommend using ensemble modeling approaches, such as biomod2, to address the limitations of individual models like MaxEnt. Detailed studies on species’ physiological responses and habitat connectivity are essential for robust forest planning under climate change.

## Figures and Tables

**Table 1:** Changes in the suitable habitat area of *B. alnoides* in different scenarios.

Period	Area (10^4^ km^2^)			Rate of Change (%)		
	Stability	Expansion	Contraction	Stability	Expansion	Contraction
2050s-SSP126	136.22	15.71	82.60	58.08	6.70	35.22
2070s-SSP126	119.92	15.26	98.98	51.21	6.52	42.27
2090s-SSP126	128.04	24.21	90.69	52.7	9.97	37.33
2050s-SSP370	138.80	20.55	79.95	58.00	8.59	33.41
2070s-SSP370	115.76	20.86	103.25	48.26	8.70	43.04
2090s-SSP370	102.59	17.18	116.25	43.47	7.28	49.25
2050s-SSP585	143.69	21.85	75.09	59.72	9.08	31.21
2070s-SSP585	100.54	19.63	118.32	42.16	8.23	49.61
2090s-SSP585	102.02	19.08	116.88	42.87	8.02	49.11

## Data Availability

Location records and environmental variables have been added to the Supplementary Materials.

## Supplementary Materials

The following supporting information can be downloaded at: https://www.mdpi.com/article/10.3390/plants14162539/s1, Table S1. Thirty-eight environmental variables used in this study. Table S2. Correlation analysis of 38 environmental variables. Table S3. Seventeen environmental factors predicting potential distribution of *Betula alnoides* in this study.
